# Микроцефальная остеодиспластическая примордиальная карликовость II типа (МОПК II типа): описание клинического случая

**DOI:** 10.14341/probl13517

**Published:** 2025-07-22

**Authors:** Н. А. Макрецкая, Н. Ю. Калинченко, А. Н. Тюльпаков

**Affiliations:** Медико-генетический научный центр им. академика Н.П. Бочкова; Национальный медицинский исследовательский центр эндокринологии им. академика И.И. Дедова; Медико-генетический научный центр им. академика Н.П. Бочкова; Российская детская клиническая больница

**Keywords:** микроцефальная остеодиспластическая примордиальная карликовость II типа, ЗВУР, низкорослость, PCNT

## Abstract

Задержка внутриутробного развития (ЗВУР) представляет собой патологическое состояние, характеризующееся низкой массой и/или длиной плода (≤-2 SD) для данного пола и гестационного возраста. Примерно в 10% случаев ЗВУР не компенсируется в постнатальном периоде, в основе патогенеза данного состояния в таком случае лежат различные моногенные синдромы или хромосомные аномалии. Сложность в постановке патогенетического диагноза в данной группе пациентов обусловлена, с одной стороны, схожестью фенотипических проявлений в структуре ЗВУР, с другой — вариабельностью клинических проявлений в рамках конкретного синдрома. Проведение различных молекулярно-генетических исследований является основным методом диагностики формы ЗВУР. Одним из наиболее распространенных наследственных вариантов задержки внутриутробного развития является микроцефальная остеодиспластическая примордиальная карликовость II типа (МОПК II типа), фенотипическими особенностями которой являются наличие скелетных аномалий и цереброваскулярных изменений. Заболевание обусловлено наличием биаллельных мутаций в гене PCNT. В данной работе представлена клиническая характеристика первого пациента с микроцефальной остеодиспластической примордиальной карликовостью II типа в Российской Федерации. Нуклеотидные изменения, выявленные у пациента, ранее не описаны в мировой литературе.

## Abstract (en)

Small for gestational age (SGA) refers to the size of an infant at birth, and is defined as a birth weight and/or birth length below the −2.0 SDS for the gestational age. In approximately 10% of cases, SGA is not compensated for in the postnatal period, with the pathogenesis of this condition being attributed to various monogenic syndromes or chromosomal abnormalities. The difficulty in making a pathogenetic diagnosis in this group of patients is due, on the one hand, to the similarity of phenotypic manifestations in the structure of the disease, on the other hand, to the variability of clinical manifestations within a specific syndrome. Conducting various molecular genetic studies is the main method of diagnosing the form of SGA. Microcephalic osteodysplastic primordial dwarfism type II (MOPD II) is one of the most common genetic variants of SGA, with its phenotypic features including skeletal anomalies and cerebrovascular changes. The disease is caused by biallelic mutations in PCNT gene. This study presents the clinical characteristics of the first patient with microcephalic osteodysplastic primordial dwarfism type II in the Russian Federation. The nucleotide changes detected in the patient have not been previously described in the world literature.

## АКТУАЛЬНОСТЬ

Задержка внутриутробного развития (ЗВУР) — состояние, характеризующееся отставанием в массе и/или длине плода на два и более стандартных отклонений (SD) (или ниже 3-го перцентиля) для данного гестационного возраста и пола [1]. Причины развития ЗВУР многофакторны и включают образ жизни матери, акушерские нарушения, эпигенетичекие и генетические нарушения. Примерно в 10% случаев у детей, рожденных со ЗВУР, не происходит нормализации показателей роста и веса к возрасту 2–3 лет [1][2]. В таких случаях важнейшим шагом в диагностике становится установка патогенетического диагноза для определения дальнейшей тактики лечения и наблюдения за данными пациентами. В частности, решение вопроса о целесообразности назначения терапии гормоном роста. Различные методы молекулярно-генетических исследований на сегодняшний день вышли на первый план в диагностике причин развития ЗВУР.

Среди моногенных вариантов ЗВУР отдельно выделена группа генетических дефектов, приводящих к нарушению фундаментальных клеточных процессов, которые затрагивают не только зоны роста, но и множество других тканей по всему организму. Одним из вариантов, входящих в данную группу, является микроцефальная остеодиспластическая примордиальная карликовость II типа (МОПК II типа) (OMIM #210720). Заболевание с аутосомно-рецессивным механизмом наследования, характерными проявлениями которого являются тяжелая задержка внутриутробного и постнатального развития с микроцефалией, костными аномалиями, а также сосудистыми изменениями ЦНС у ряда пациентов, обуславливающими ранние инсульты. Молекулярный механизм, приводящий к формированию МОПК II типа, — патогенные варианты в гене PCNT [3–5].

В настоящей работе представлено первое описание клинического случая микроцефальной остеодиспластической примордиальной карликовости II типа в Российской Федерации.

## ОПИСАНИЕ СЛУЧАЯ

Мальчик от второй беременности (1-я — медикаментозный аборт), протекавшей на фоне носительства вируса простого герпеса, цитомегаловируса, наличие вредных привычек мать отрицает. Возраст матери на момент беременности — 29 лет. Во второй половине беременности диагностирована ЗВУР плода. Роды самостоятельные на 36-й неделе, при рождении масса — 1500 г (-3,3 SD), длина — 41 см (-3,1 SD), масса-ростовой коэффициент — 37 (N: 60-80), оценка по шкале Апгар 5/6. Наследственность не отягощена, близкородственный брак родители отрицают, этническая принадлежность — русские. Рост матери — 167 см, отца — 180 см, целевой рост — 180 см (0,8 SD), рассчитан с помощью приложения Auxology.

В возрасте 1 года рост ребенка составлял 61,5 см (-5,5 SD), исследован гормональный профиль: ИФР-1 — 159 нг/мл (N: 17,0–95,0), ТТГ — 1,4 мМЕ/мл (N: 0,5–5,0), свТ4 — 18,6 пмоль/л (N: 11,4–19,5), кортизол — 350,8 нмоль/л (N: 77,0–630,0), пролактин — 1158 мМЕ/л (N: 60,0–510,0). По данным МРТ головного мозга диагностирована наружная гидроцефалия.

В 3,8 года пациент впервые обследован в отделении эндокринологии: рост — 82 см (-4,3 SD), клинически отмечается микроцефалия, клювовидный нос, монголоидный разрез глаз, низкопосаженные уши, микрогнатия, гиперпигментированные пятна цвета «кофе с молоком» в области груди и шеи. Уровень ИФР-1 составил 172,3 нг/мл (N: 31–175). Учитывая выраженное отставание в росте, принято решение о проведении СТГ-стимуляционной пробы с клонидином: максимальный выброс СТГ составил 10,1 нг/мл, что исключает наличие у ребенка СТГ-дефицита [6]. В возрасте 6 лет: рост — 97 см (-4,1 SD), окружность головы — 43,5 см (-5,9 SD), по данным МРТ головного мозга патологических изменений не выявлено. Динамически ребенок обследован в возрасте 10 лет: рост — 111,5 см (-4,2 SD), вес — 20 кг (SDS ИМТ -0,5), верхний сегмент — 59 см (-4,7 SD), нижний сегмент — 52,5 см (-2,8 SD). Скорость роста — 4,3 см/год. Данные динамики роста представлены на рисунке 1. Половое развитие — Таннер 2: G2P1, яички в мошонке d=s по 3 мл. В общем анализе крови диагностирован тромбоцитоз до 382х10^9 кл/л (N: 148–339х10^9 кл/л). В гормональном профиле уровень ИФР-1 — 316,8 нг/мл (N: 23,0–459,0), ЛГ — 0,4 Ед/л (N: 0–1,5), ФСГ — 2,4 Ед/л (N: 0–2,0), тестостерон — 0,7 нмоль/л (N: 0,3–2,3). Костный возраст соответствует 10,5 года по атласу TW20. Проведена проба с аналогами гонадотропин-релизинг-гормона (ГнРГ) с целью подтверждения дебюта пубертата, максимальный выброс ЛГ составил 18,3 Ед/л, что соответствует пубертатным значениям. У офтальмолога диагностирован смешанный астигматизм. Нарушений со стороны других органов и систем не выявлено. Учитывая клинические данные пациента, проведено молекулярно-генетическое исследование методом полноэкзомного секвенирования, в гене PCNT (NM_006031.5) выявлено два варианта нуклеотидной последовательности: p.Asp2452fs (c.7354delG) и c.3464+5G>A.

**Figure:**
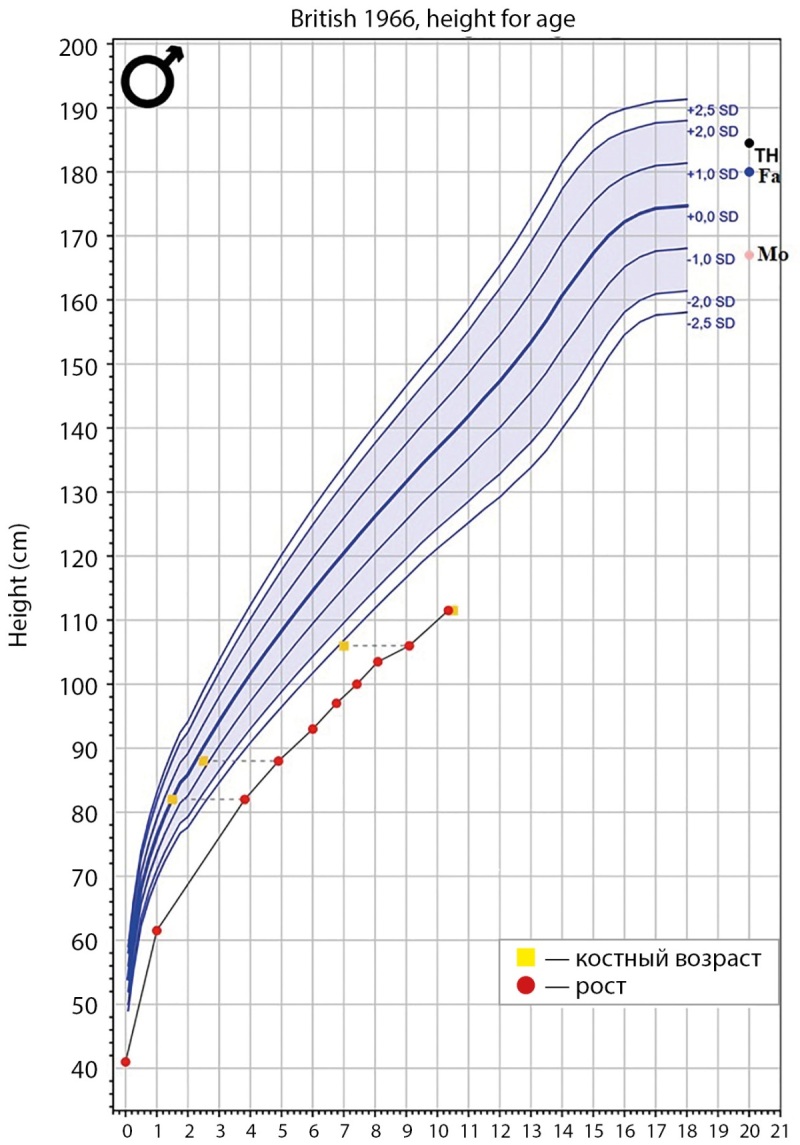
Рисунок 1. Кривая роста пациента с указанием данных роста и костного возраста. Примечания: ось абсцисс — возраст пробанда, года; ось ординат — рост пробанда, см; Fa — рост отца; Mo — рост матери; TH — целевой рост пробанда (рассчитан в программе Growth analyser).

## ОБСУЖДЕНИЕ

Впервые МОПК II типа описана в 1982 г. Majewski Ranke и Schinze у 3 неродственных сибсов как новый вариант примордиальной карликовости [3]. Отличительной особенностью данного варианта было наличие костных аномалий: диспропорциональные укорочения предплечий и нижних конечностей в раннем возрасте; брахимезофалангия; брахиметакарпия; V-образное расширение дистальных метафизов и треугольная форма дистальных эпифизов бедренной кости; проксимальный эпифизеолиз бедренной кости и тазобедренного сустава.

Только в 2008 г. одновременно двумя коллективами Rauch и соавт. и Griffith и соавт. установлено, что биаллельные мутации в гене PCNT являются причиной развития МОПК II типа [4][5]. Ген картирован на хромосоме 21q22.3 и содержит 47 экзомов [4]. Белок PCNT является высококонсервативным, состоит из 3336 аминокислотных остатков и встречается в тканях человека повсеместно [7]. Данный белок является основным компонентом перицентриолярного вещества и играет ключевую роль в структуре и функции центросом. Дефицит белка PCNT приводит к аномалии центросом, приводящей к нарушению сборки и дезориентации веретена, неправильной сегрегации хромосом, митотической недостаточности и нарушению прогрессирования клеточного цикла [8–9].

Анализ литературы с клиническими данными пациентов позволил систематизировать фенотипические особенности заболевания. Для пренатального периода характерно формирование ранней ЗВУР, примерно между 12-й и 14-й неделями. Масса новорожденных варьировала от 450 до 1600 г, при этом стоит отметить, что средний срок родов составлял 35 недель. Также отмечались низкие показатели длины при рождении от 30 до 40 см. Окружность головы при рождении колебалась от 22 до 29 см, что соответствовало микроцефалии, однако визуально голова выглядела пропорционально размерам тела. В постнатальном периоде отмечается нарастание диспропорций относительно окружности головы и длины тела. Костный возраст как правило отстает от паспортного на 2–5 лет. При этом авторы работ уточняют, что наличие скелетной дисплазии может исказить истинные показатели данного параметра, в частности возраст прорезывания зубов свидетельствует об ускорении созревания костной ткани [10]. Кроме того, для пациентов с МОПК II типа характерно развитие преждевременного полового развития, особенно у девочек (средний возраст телархе — 7 лет, менархе — 9 лет). Медиана конечного роста данных пациентов составила 100 см, а остановка роста в среднем приходится на паспортный возраст 12 лет. Около 50% пациентов получали терапию рекомбинантным гормоном роста, однако эффекта получено не было [10][11].

Черепно-лицевые особенности, отличающие МОПК II типа от других синдромов со ЗВУР, это выступающий нос с широкой переносицей и корнем; низкопосаженные уши, часто без мочки, пропорциональные размерам головы; мелкие зубы с гипоплазированной эмалью, в ряде случаев отмечается гипоплазия/аплазия корней [10][12]. Кроме того, для данных пациентов характерно развитие дальнозоркости или астигматизма в возрасте от 3 до 5 лет [10]. Важным диагностическим критерием является формирование пятен цвета «кофе с молоком» в среднем к возрасту двух лет. К 5 годам описано усиление пигментации в области шеи и подмышек [10]. Учитывая наличие характерных для резистентности к инсулину гиперпигментаций, в 2011 году Huang-Doran и соавт. обследован 21 пациент с МОПК II типа: в 81% случаев (18/21) диагностирована инсулинорезистентность, а в 48% — сахарный диабет (средний возраст дебюта — 15 лет (5–28)) [13].

Главным жизнеугрожающим состоянием, характерным для МОПК II типа, являются сосудистые изменения. В 20–59% случаев диагностированы изменения ЦНС, такие как болезнь мойя-мойя или множественные церебральные аневризмы, которые могут приводить к ранним инсультам [10][11][14–16]. У 17% пациентов отмечались ранние инфаркты миокарда (Ме 24 года), в 28% случаев выявлены ДМПП, ДМЖП или открытое овальное окно [14]. Аномалии почечных артерий выявлены в 32% случаев: 28% — добавочные почечные артерии, 4% — аневризмы [14].

В общем анализе крови у пациентов с МОПК II типа диагностирован бессимптомный тромбоцитоз, лейкоцитоз и/или анемия [17][18].

В настоящей работе впервые для российской литературы представлены данные пациента с МОПК II типа. У пациента выявлены две не описанные ранее мутации в гене PCNT (NM_006031.5): вариант в экзоне 34 p.Asp2452fs, приводящий к сдвигу рамки считывая, начиная с кодона 2452, и вариант в интроне 17 c.3464+5G>A. Алгоритм предсказания влияния мутаций SpliceAI расценивает последний вариант как вызывающий нарушение сплайсинга. Данные варианты нуклеотидной последовательности отсутствуют в базе популяционных частот gnomAD. В соответствии с критериями, используемыми для интерпретации результатов секвенирования [19][20], оба варианта оценены как вероятно патогенные (PM2, PVS1, PP3, PP4). Важно отметить, что клинически у нашего пациента не диагностировано фенотипических особенностей, характерных для МОПК II типа, в частности сосудистых изменений и нарушений углеводного обмена, что может быть связано, во-первых, с вариабельностью данных признаков, во-вторых, с возрастом пробанда.

Представленное описание клинического случая демонстрирует сложность проведения дифференциальной диагностики в структуре патологии. Согласно международному консенсусу выделяют 13 нозологических форм ЗВУР с микроцефалией и 29 генов-кандидатов, лежащих в основе их патогенеза [1]. Таким образом, молекулярно-генетическая диагностика является основополагающим этапом постановки диагноза.

## ЗАКЛЮЧЕНИЕ

Нами представлено первое для отечественной литературы описание клинического случая микроцефальной остеодиспластической примордиальной карликовости II типа. Результаты настоящей работы демонстрируют важность проведения молекулярно-генетических исследований пациентам из данной группы заболеваний. Определение молекулярной природы позволяет скорректировать тактику наблюдения и обследования пациентов.

## ДОПОЛНИТЕЛЬНАЯ ИНФОРМАЦИЯ

Источники финансирования. Работа выполнена в рамках государственного задания Минобрнауки России для ФГБНУ «МГНЦ».

Конфликт интересов. Авторы декларируют отсутствие явных и потенциальных конфликтов интересов, связанных с содержанием настоящей статьи.

Участие авторов. Все авторы одобрили финальную версию статьи перед публикацией, выразили согласие нести ответственность за все аспекты работы, подразумевающую надлежащее изучение и решение вопросов, связанных с точностью или добросовестностью любой части работы.

Согласие пациента. Добровольное информированное согласие законных представителей пациента на публикацию в журнале «Проблемы эндокринологии» получено.
